# Supplementary material for: Use of signals of positive and negative selection to distinguish cancer genes and passenger genes
Source: eLife. 2021 Jan 11;10:e59629. doi: 10.7554/eLife.59629 (PMC7877913; doi:10.7554/eLife.59629)
Supplement: Supplementary file 16. [file elife-59629-supp16.docx]

**Supplementary file 16**

**Expected fractions of nonsense, missense and silent substitutions of codons in the absence of selection assuming that C>A, C>G, C>T, T>A, T>C and T>G mutations occur with equal probability**

**Amino acid mutation Expected fraction**

**Nonsense, fN** 0.04189 ± 0.07075

**Missense, fM**  0.71403 ± 0.11558

**Silent, fS**  0.24408 ± 0.11970

The expected fractions of nonsense, missense and silent amino acid mutations of individual codons are shown in Supplementary files 14 and 15.
