# Supplementary material for: Use of signals of positive and negative selection to distinguish cancer genes and passenger genes
Source: eLife. 2021 Jan 11;10:e59629. doi: 10.7554/eLife.59629 (PMC7877913; doi:10.7554/eLife.59629)
Supplement: Supplementary file 18. [file elife-59629-supp18.docx]

# Supplementary file 18

**Expected fractions of nonsense, missense and silent substitutions of various codons in the absence of selection assuming that only *C>A and G>T*** ***mutations* occur**

**C>A and G>T** **mutations only**

WTC, Wild Type Codon; WTAA, Wild Type Amino Acid; WTN, Wild Type Nucleotide; MN, Mutant Nucleotide; MC, mutant codon; MAA, mutant amino acid;

Category of mutation: NON, nonsense; MIS, missense; SIL, silent);

Fraction M, fraction of missense mutations; Fraction S, fraction of silent mutations; Fraction N, fraction of nonsense mutations;

|  | **WTC** | **WTAA** | **WTN** | **MN** | **MC** | **MAA** | **Category** | **Fraction M** | **Fraction S** | **Fraction N** |
| --- | --- | --- | --- | --- | --- | --- | --- | --- | --- | --- |
|  | AAA | Lys | A | T | TAA | STOP | NON | 0.0 | 0.0 | 0.0 |
|  | AAA | Lys | A | C | CAA | Gln | MIS |  |  |  |
|  | AAA | Lys | A | G | GAA | Glu | MIS |  |  |  |
|  | AAA | Lys | A | G | AGA | Arg | MIS |  |  |  |
|  | AAA | Lys | A | C | ACA | Thr | MIS |  |  |  |
|  | AAA | Lys | A | T | ATA | Ile | MIS |  |  |  |
|  | AAA | Lys | A | C | AAC | Asn | MIS |  |  |  |
|  | AAA | Lys | A | T | AAT | Asn | MIS |  |  |  |
|  | AAA | Lys | A | G | AAG | Lys | SIL |  |  |  |
|  | AAC | Asn | A | C | CAC | His | MIS | 1.0 | 0.0 | 0.0 |
|  | AAC | Asn | A | G | GAC | Asp | MIS |  |  |  |
|  | AAC | Asn | A | T | TAC | Tyr | MIS |  |  |  |
|  | AAC | Asn | A | C | ACC | Thr | MIS |  |  |  |
|  | AAC | Asn | A | G | AGC | Ser | MIS |  |  |  |
|  | AAC | Asn | A | T | ATC | Ile | MIS |  |  |  |
|  | ***AAC*** | ***Asn*** | ***C*** | ***A*** | ***AAA*** | ***Lys*** | ***MIS*** |  |  |  |
|  | AAC | Asn | C | G | AAG | Lys | MIS |  |  |  |
|  | AAC | Asn | C | T | AAT | Asn | SIL |  |  |  |
|  | AAG | Lys | A | T | TAG | STOP | NON | 1.0 | 0.0 | 0.0 |
|  | AAG | Lys | A | C | CAG | Gln | MIS |  |  |  |
|  | AAG | Lys | A | G | GAG | Glu | MIS |  |  |  |
|  | AAG | Lys | A | G | AGG | Arg | MIS |  |  |  |
|  | AAG | Lys | A | C | ACG | Thr | MIS |  |  |  |
|  | AAG | Lys | A | T | ATG | Met | MIS |  |  |  |
|  | AAG | Lys | G | C | AAC | Asn | MIS |  |  |  |
|  | ***AAG*** | ***Lys*** | ***G*** | ***T*** | ***AAT*** | ***Asn*** | ***MIS*** |  |  |  |
|  | AAG | Lys | G | A | AAA | Lys | SIL |  |  |  |
|  |  |  |  |  |  |  |  |  |  |  |

|  | AAT | Asn | A | C | CAT | His | MIS | 0.0 | 0.0 | 0.0 |
| --- | --- | --- | --- | --- | --- | --- | --- | --- | --- | --- |
|  | AAT | Asn | A | G | GAT | Asp | MIS |  |  |  |
|  | AAT | Asn | A | T | TAT | Tyr | MIS |  |  |  |
|  | AAT | Asn | A | C | ACT | Thr | MIS |  |  |  |
|  | AAT | Asn | A | G | AGT | Ser | MIS |  |  |  |
|  | AAT | Asn | A | T | ATT | Ile | MIS |  |  |  |
|  | AAT | Asn | T | A | AAA | Lys | MIS |  |  |  |
|  | AAT | Asn | T | G | AAG | Lys | MIS |  |  |  |
|  | AAT | Asn | T | C | AAC | Asn | SIL |  |  |  |
|  | ACA | Thr | A | C | CCA | Pro | MIS | 1.0 | 0.0 | 0.0 |
|  | ACA | Thr | A | G | GCA | Ala | MIS |  |  |  |
|  | ACA | Thr | A | T | TCA | Ser | MIS |  |  |  |
|  | ***ACA*** | ***Thr*** | ***C*** | ***A*** | ***AAA*** | ***Lys*** | ***MIS*** |  |  |  |
|  | ACA | Thr | C | G | AGA | Arg | MIS |  |  |  |
|  | ACA | Thr | C | T | ATA | Ile | MIS |  |  |  |
|  | ACA | Thr | A | C | ACC | Thr | SIL |  |  |  |
|  | ACA | Thr | A | G | ACG | Thr | SIL |  |  |  |
|  | ACA | Thr | A | T | ACT | Thr | SIL |  |  |  |
|  | ACC | Thr | A | C | CCC | Pro | MIS | 0.5 | 0.5 | 0.0 |
|  | ACC | Thr | A | G | GCC | Ala | MIS |  |  |  |
|  | ACC | Thr | A | T | TCC | Ser | MIS |  |  |  |
|  | ACC | Thr | C | G | AGC | Ser | MIS |  |  |  |
|  | ***ACC*** | ***Thr*** | ***C*** | ***A*** | ***AAC*** | ***Asn*** | ***MIS*** |  |  |  |
|  | ACC | Thr | C | T | ATC | Ile | MIS |  |  |  |
|  | ***ACC*** | ***Thr*** | ***C*** | ***A*** | ***ACA*** | ***Thr*** | ***SIL*** |  |  |  |
|  | ACC | Thr | C | G | ACG | Thr | SIL |  |  |  |
|  | ACC | Thr | C | T | ACT | Thr | SIL |  |  |  |
|  | ACG | Thr | A | C | CCG | Pro | MIS | 0.5 | 0.5 | 0.0 |
|  | ACG | Thr | A | G | GCG | Ala | MIS |  |  |  |
|  | ACG | Thr | A | T | TCG | Ser | MIS |  |  |  |
|  | ***ACG*** | ***Thr*** | ***C*** | ***A*** | ***AAG*** | ***Lys*** | ***MIS*** |  |  |  |
|  | ACG | Thr | C | G | AGG | Arg | MIS |  |  |  |
|  | ACG | Thr | C | T | ATG | Met | MIS |  |  |  |
|  | ACG | Thr | G | A | ACA | Thr | SIL |  |  |  |
|  | ACG | Thr | G | C | ACC | Thr | SIL |  |  |  |
|  | ***ACG*** | ***Thr*** | ***G*** | ***T*** | ***ACT*** | ***Thr*** | ***SIL*** |  |  |  |
|  | ACT | Thr | A | C | CCT | Pro | MIS | 1.0 | 0.0 | 0.0 |
|  | ACT | Thr | A | G | GCT | Ala | MIS |  |  |  |
|  | ACT | Thr | A | T | TCT | Ser | MIS |  |  |  |
|  | ACT | Thr | C | G | AGT | Ser | MIS |  |  |  |
|  | ***ACT*** | ***Thr*** | ***C*** | ***A*** | ***AAT*** | ***Asn*** | ***MIS*** |  |  |  |
|  | ACT | Thr | C | T | ATT | Ile | MIS |  |  |  |
|  | ACT | Thr | T | A | ACA | Thr | SIL |  |  |  |
|  | ACT | Thr | T | C | ACC | Thr | SIL |  |  |  |
|  | ACT | Thr | T | G | ACG | Thr | SIL |  |  |  |

|  |  |  |  |  |  |  |  |  |  |  |
| --- | --- | --- | --- | --- | --- | --- | --- | --- | --- | --- |
|  | AGA | Arg | A | T | TGA | STOP | NON | 1.0 | 0.0 | 0.0 |
|  | AGA | Arg | A | G | GGA | Gly | MIS |  |  |  |
|  | AGA | Arg | A | C | CGA | Arg | SIL |  |  |  |
|  | AGA | Arg | G | A | AAA | Lys | MIS |  |  |  |
|  | AGA | Arg | G | C | ACA | Thr | MIS |  |  |  |
|  | ***AGA*** | ***Arg*** | ***G*** | ***T*** | ***ATA*** | ***Ile*** | ***MIS*** |  |  |  |
|  | AGA | Arg | A | C | AGC | Ser | MIS |  |  |  |
|  | AGA | Arg | A | T | AGT | Ser | MIS |  |  |  |
|  | AGA | Arg | A | G | AGG | Arg | SIL |  |  |  |
|  | AGC | Ser | A | G | GGC | Gly | MIS | 1.0 | 0.0 | 0.0 |
|  | AGC | Ser | A | C | CGC | Arg | MIS |  |  |  |
|  | AGC | Ser | A | T | TGC | Cys | MIS |  |  |  |
|  | AGC | Ser | G | C | ACC | Thr | MIS |  |  |  |
|  | AGC | Ser | G | A | AAC | Asn | MIS |  |  |  |
|  | ***AGC*** | ***Ser*** | ***G*** | ***T*** | ***ATC*** | ***Ile*** | ***MIS*** |  |  |  |
|  | ***AGC*** | ***Ser*** | ***C*** | ***A*** | ***AGA*** | ***Arg*** | ***MIS*** |  |  |  |
|  | AGC | Ser | C | G | AGG | Arg | MIS |  |  |  |
|  | AGC | Ser | C | T | AGT | Ser | SIL |  |  |  |
|  | AGG | Arg | A | G | GGG | Gly | MIS | 1.0 | 0.0 | 0.0 |
|  | AGG | Arg | A | T | TGG | Trp | MIS |  |  |  |
|  | AGG | Arg | A | C | CGG | Arg | SIL |  |  |  |
|  | AGG | Arg | G | A | AAG | Lys | MIS |  |  |  |
|  | AGG | Arg | G | C | ACG | Thr | MIS |  |  |  |
|  | ***AGG*** | ***Arg*** | ***G*** | ***T*** | ***ATG*** | ***Met*** | ***MIS*** |  |  |  |
|  | AGG | Arg | G | C | AGC | Ser | MIS |  |  |  |
|  | ***AGG*** | ***Arg*** | ***G*** | ***T*** | ***AGT*** | ***Ser*** | ***MIS*** |  |  |  |
|  | AGG | Arg | G | A | AGA | Arg | SIL |  |  |  |
|  | AGT | Ser | A | G | GGT | Gly | MIS | 1.0 | 0.0 | 0.0 |
|  | AGT | Ser | A | C | CGT | Arg | MIS |  |  |  |
|  | AGT | Ser | A | T | TGT | Cys | MIS |  |  |  |
|  | AGT | Ser | G | C | ACT | Thr | MIS |  |  |  |
|  | AGT | Ser | G | A | AAT | Asn | MIS |  |  |  |
|  | ***AGT*** | ***Ser*** | ***G*** | ***T*** | ***ATT*** | ***Ile*** | ***MIS*** |  |  |  |
|  | AGT | Ser | T | A | AGA | Arg | MIS |  |  |  |
|  | AGT | Ser | T | G | AGG | Arg | MIS |  |  |  |
|  | AGT | Ser | T | C | AGC | Ser | SIL |  |  |  |
|  | ATA | Ile | A | C | CTA | Leu | MIS | 0.0 | 0.0 | 0.0 |
|  | ATA | Ile | A | G | GTA | Val | MIS |  |  |  |
|  | ATA | Ile | A | T | TTA | Leu | MIS |  |  |  |
|  | ATA | Ile | T | A | AAA | Lys | MIS |  |  |  |
|  | ATA | Ile | T | C | ACA | Thr | MIS |  |  |  |
|  | ATA | Ile | T | G | AGA | Arg | MIS |  |  |  |
|  | ATA | Ile | A | G | ATG | Met | MIS |  |  |  |
|  | ATA | Ile | A | C | ATC | Ile | SIL |  |  |  |
|  | ATA | Ile | A | T | ATT | Ile | SIL |  |  |  |

|  | ATC | Ile | A | C | CTC | Leu | MIS | 0.0 | 1.0 | 0.0 |
| --- | --- | --- | --- | --- | --- | --- | --- | --- | --- | --- |
|  | ATC | Ile | A | G | GTC | Val | MIS |  |  |  |
|  | ATC | Ile | A | T | TTC | Phe | MIS |  |  |  |
|  | ATC | Ile | T | A | AAC | Asn | MIS |  |  |  |
|  | ATC | Ile | T | C | ACC | Thr | MIS |  |  |  |
|  | ATC | Ile | T | G | AGC | Ser | MIS |  |  |  |
|  | ATC | Ile | C | G | ATG | Met | MIS |  |  |  |
|  | ***ATC*** | ***Ile*** | ***C*** | ***A*** | ***ATA*** | ***Ile*** | ***SIL*** |  |  |  |
|  | ATC | Ile | C | T | ATT | Ile | SIL |  |  |  |
|  | ATG | Met | A | C | CTG | Leu | MIS | 1.0 | 0.0 | 0.0 |
|  | ATG | Met | A | G | GTG | Val | MIS |  |  |  |
|  | ATG | Met | A | T | TTG | Leu | MIS |  |  |  |
|  | ATG | Met | T | A | AAG | Lys | MIS |  |  |  |
|  | ATG | Met | T | C | ACG | Thr | MIS |  |  |  |
|  | ATG | Met | T | G | AGG | Arg | MIS |  |  |  |
|  | ATG | Met | G | A | ATA | Ile | MIS |  |  |  |
|  | ATG | Met | G | C | ATC | Ile | MIS |  |  |  |
|  | ***ATG*** | ***Met*** | ***G*** | ***T*** | ***ATT*** | ***Ile*** | ***MIS*** |  |  |  |
|  | ATT | Ile | A | C | CTT | Leu | MIS | 0.0 | 0.0 | 0.0 |
|  | ATT | Ile | A | G | GTT | Val | MIS |  |  |  |
|  | ATT | Ile | A | T | TTT | Phe | MIS |  |  |  |
|  | ATT | Ile | T | A | AAT | Asn | MIS |  |  |  |
|  | ATT | Ile | T | C | ACT | Thr | MIS |  |  |  |
|  | ATT | Ile | T | G | AGT | Ser | MIS |  |  |  |
|  | ATT | Ile | T | G | ATG | Met | MIS |  |  |  |
|  | ATT | Ile | T | A | ATA | Ile | SIL |  |  |  |
|  | ATT | Ile | T | C | ATC | Ile | SIL |  |  |  |
|  | CAA | Gln | C | T | TAA | STOP | NON | 1.0 | 0.0 | 0.0 |
|  | ***CAA*** | ***Gln*** | ***C*** | ***A*** | ***AAA*** | ***Lys*** | ***MIS*** |  |  |  |
|  | CAA | Gln | C | G | GAA | Glu | MIS |  |  |  |
|  | CAA | Gln | A | C | CCA | Pro | MIS |  |  |  |
|  | CAA | Gln | A | G | CGA | Arg | MIS |  |  |  |
|  | CAA | Gln | A | T | CTA | Leu | MIS |  |  |  |
|  | CAA | Gln | A | C | CAC | His | MIS |  |  |  |
|  | CAA | Gln | A | T | CAT | His | MIS |  |  |  |
|  | CAA | Gln | A | G | CAG | Gln | SIL |  |  |  |
|  | ***CAC*** | ***His*** | ***C*** | ***A*** | ***AAC*** | ***Asn*** | ***MIS*** | 1.0 | 0.0 | 0.0 |
|  | CAC | His | C | G | GAC | Asp | MIS |  |  |  |
|  | CAC | His | C | T | TAC | Tyr | MIS |  |  |  |
|  | CAC | His | A | G | CGC | Arg | MIS |  |  |  |
|  | CAC | His | A | C | CCC | Pro | MIS |  |  |  |
|  | CAC | His | A | T | CTC | Leu | MIS |  |  |  |
|  | ***CAC*** | ***His*** | ***C*** | ***A*** | ***CAA*** | ***Gln*** | ***MIS*** |  |  |  |
|  | CAC | His | C | G | CAG | Gln | MIS |  |  |  |
|  | CAC | His | C | T | CAT | His | SIL |  |  |  |

|  | CAG | Gln | C | T | TAG | STOP | NON | 1.0 | 0.0 | 0.0 |
| --- | --- | --- | --- | --- | --- | --- | --- | --- | --- | --- |
|  | ***CAG*** | ***Gln*** | ***C*** | ***A*** | ***AAG*** | ***Lys*** | ***MIS*** |  |  |  |
|  | CAG | Gln | C | G | GAG | Glu | MIS |  |  |  |
|  | CAG | Gln | A | C | CCG | Pro | MIS |  |  |  |
|  | CAG | Gln | A | G | CGG | Arg | MIS |  |  |  |
|  | CAG | Gln | A | T | CTG | Leu | MIS |  |  |  |
|  | CAG | Gln | G | C | CAC | His | MIS |  |  |  |
|  | ***CAG*** | ***Gln*** | ***G*** | ***T*** | ***CAT*** | ***His*** | ***MIS*** |  |  |  |
|  | CAG | Gln | G | A | CAA | Gln | SIL |  |  |  |
|  | ***CAT*** | ***His*** | ***C*** | ***A*** | ***AAT*** | ***Asn*** | ***MIS*** | 1.0 | 0.0 | 0.0 |
|  | CAT | His | C | G | GAT | Asp | MIS |  |  |  |
|  | CAT | His | C | T | TAT | Tyr | MIS |  |  |  |
|  | CAT | His | A | G | CGT | Arg | MIS |  |  |  |
|  | CAT | His | A | C | CCT | Pro | MIS |  |  |  |
|  | CAT | His | A | T | CTT | Leu | MIS |  |  |  |
|  | CAT | His | T | A | CAA | Gln | MIS |  |  |  |
|  | CAT | His | T | G | CAG | Gln | MIS |  |  |  |
|  | CAT | His | T | C | CAC | His | SIL |  |  |  |
|  | ***CCA*** | ***Pro*** | ***C*** | ***A*** | ***ACA*** | ***Thr*** | ***MIS*** | 1.0 | 0.0 | 0.0 |
|  | CCA | Pro | C | T | TCA | Ser | MIS |  |  |  |
|  | CCA | Pro | C | G | GCA | Ala | MIS |  |  |  |
|  | ***CCA*** | ***Pro*** | ***C*** | ***A*** | ***CAA*** | ***Gln*** | ***MIS*** |  |  |  |
|  | CCA | Pro | C | G | CGA | Arg | MIS |  |  |  |
|  | CCA | Pro | C | T | CTA | Leu | MIS |  |  |  |
|  | CCA | Pro | A | C | CCC | Pro | SIL |  |  |  |
|  | CCA | Pro | A | G | CCG | Pro | SIL |  |  |  |
|  | CCA | Pro | A | T | CCT | Pro | SIL |  |  |  |
|  | ***CCC*** | ***Pro*** | ***C*** | ***A*** | ***ACC*** | ***Thr*** | ***MIS*** | 0.66 | 0.33 | 0.0 |
|  | CCC | Pro | C | T | TCC | Ser | MIS |  |  |  |
|  | CCC | Pro | C | G | GCC | Ala | MIS |  |  |  |
|  | ***CCC*** | ***Pro*** | ***C*** | ***A*** | ***CAC*** | ***His*** | ***MIS*** |  |  |  |
|  | CCC | Pro | C | G | CGC | Arg | MIS |  |  |  |
|  | CCC | Pro | C | T | CTC | Leu | MIS |  |  |  |
|  | ***CCC*** | ***Pro*** | ***C*** | ***A*** | ***CCA*** | ***Pro*** | ***SIL*** |  |  |  |
|  | CCC | Pro | C | G | CCG | Pro | SIL |  |  |  |
|  | CCC | Pro | C | T | CCT | Pro | SIL |  |  |  |
|  | ***CCG*** | ***Pro*** | ***C*** | ***A*** | ***ACG*** | ***Thr*** | ***MIS*** | 0.66 | 0.33 | 0.0 |
|  | CCG | Pro | C | T | TCG | Ser | MIS |  |  |  |
|  | CCG | Pro | C | G | GCG | Ala | MIS |  |  |  |
|  | ***CCG*** | ***Pro*** | ***C*** | ***A*** | ***CAG*** | ***Gln*** | ***MIS*** |  |  |  |
|  | CCG | Pro | C | G | CGG | Arg | MIS |  |  |  |
|  | CCG | Pro | C | T | CTG | Leu | MIS |  |  |  |
|  | CCG | Pro | G | A | CCA | Pro | SIL |  |  |  |
|  | CCG | Pro | G | C | CCC | Pro | SIL |  |  |  |
|  | ***CCG*** | ***Pro*** | ***G*** | ***T*** | ***CCT*** | ***Pro*** | ***SIL*** |  |  |  |

|  | ***CCT*** | ***Pro*** | ***C*** | ***A*** | ***ACT*** | ***Thr*** | ***MIS*** | 1.0 | 0.0 | 0.0 |
| --- | --- | --- | --- | --- | --- | --- | --- | --- | --- | --- |
|  | CCT | Pro | C | T | TCT | Ser | MIS |  |  |  |
|  | CCT | Pro | C | G | GCT | Ala | MIS |  |  |  |
|  | ***CCT*** | ***Pro*** | ***C*** | ***A*** | ***CAT*** | ***His*** | ***MIS*** |  |  |  |
|  | CCT | Pro | C | G | CGT | Arg | MIS |  |  |  |
|  | CCT | Pro | C | T | CTT | Leu | MIS |  |  |  |
|  | CCT | Pro | T | A | CCA | Pro | SIL |  |  |  |
|  | CCT | Pro | T | C | CCC | Pro | SIL |  |  |  |
|  | CCT | Pro | T | G | CCG | Pro | SIL |  |  |  |
|  | CGA | Arg | C | T | TGA | STOP | NON | 0.5 | 0.5 | 0.0 |
|  | CGA | Arg | C | G | GGA | Gly | MIS |  |  |  |
|  | ***CGA*** | ***Arg*** | ***C*** | ***A*** | ***AGA*** | ***Arg*** | ***SIL*** |  |  |  |
|  | CGA | Arg | G | A | CAA | Gln | MIS |  |  |  |
|  | CGA | Arg | G | C | CCA | Pro | MIS |  |  |  |
|  | ***CGA*** | ***Arg*** | ***G*** | ***T*** | ***CTA*** | ***Leu*** | ***MIS*** |  |  |  |
|  | CGA | Arg | A | C | CGC | Arg | SIL |  |  |  |
|  | CGA | Arg | A | G | CGG | Arg | SIL |  |  |  |
|  | CGA | Arg | A | T | CGT | Arg | SIL |  |  |  |
|  | ***CGC*** | ***Arg*** | ***C*** | ***A*** | ***AGC*** | ***Ser*** | ***MIS*** | 0.66 | 0.33 | 0.0 |
|  | CGC | Arg | C | G | GGC | Gly | MIS |  |  |  |
|  | CGC | Arg | C | T | TGC | Cys | MIS |  |  |  |
|  | CGC | Arg | G | A | CAC | His | MIS |  |  |  |
|  | CGC | Arg | G | C | CCC | Pro | MIS |  |  |  |
|  | ***CGC*** | ***Arg*** | ***G*** | ***T*** | ***CTC*** | ***Leu*** | ***MIS*** |  |  |  |
|  | ***CGC*** | ***Arg*** | ***C*** | ***A*** | ***CGA*** | ***Arg*** | ***SIL*** |  |  |  |
|  | CGC | Arg | C | G | CGG | Arg | SIL |  |  |  |
|  | CGC | Arg | C | T | CGT | Arg | SIL |  |  |  |
|  | CGG | Arg | C | G | GGG | Gly | MIS | 0.33 | 0.66 | 0.0 |
|  | CGG | Arg | C | T | TGG | Trp | MIS |  |  |  |
|  | ***CGG*** | ***Arg*** | ***C*** | ***A*** | ***AGG*** | ***Arg*** | ***SIL*** |  |  |  |
|  | CGG | Arg | G | A | CAG | Gln | MIS |  |  |  |
|  | CGG | Arg | G | C | CCG | Pro | MIS |  |  |  |
|  | ***CGG*** | ***Arg*** | ***G*** | ***T*** | ***CTG*** | ***Leu*** | ***MIS*** |  |  |  |
|  | CGG | Arg | G | A | CGA | Arg | SIL |  |  |  |
|  | CGG | Arg | G | C | CGC | Arg | SIL |  |  |  |
|  | ***CGG*** | ***Arg*** | ***G*** | ***T*** | ***CGT*** | ***Arg*** | ***SIL*** |  |  |  |
|  | ***CGT*** | ***Arg*** | ***C*** | ***A*** | ***AGT*** | ***Ser*** | ***MIS*** | 1.0 | 0.0 | 0.0 |
|  | CGT | Arg | C | G | GGT | Gly | MIS |  |  |  |
|  | CGT | Arg | C | T | TGT | Cys | MIS |  |  |  |
|  | CGT | Arg | G | A | CAT | His | MIS |  |  |  |
|  | CGT | Arg | G | C | CCT | Pro | MIS |  |  |  |
|  | ***CGT*** | ***Arg*** | ***G*** | ***T*** | ***CTT*** | ***Leu*** | ***MIS*** |  |  |  |
|  | CGT | Arg | T | A | CGA | Arg | SIL |  |  |  |
|  | CGT | Arg | T | C | CGC | Arg | SIL |  |  |  |
|  | CGT | Arg | T | G | CGG | Arg | SIL |  |  |  |

|  |  |  |  |  |  |  |  |  |  |  |
| --- | --- | --- | --- | --- | --- | --- | --- | --- | --- | --- |
|  | ***CTA*** | ***Leu*** | ***C*** | ***A*** | ***ATA*** | ***Ile*** | ***MIS*** | 1.0 | 0.0 | 0.0 |
|  | CTA | Leu | C | G | GTA | Val | MIS |  |  |  |
|  | CTA | Leu | C | T | TTA | Leu | SIL |  |  |  |
|  | CTA | Leu | T | A | CAA | Gln | MIS |  |  |  |
|  | CTA | Leu | T | C | CCA | Pro | MIS |  |  |  |
|  | CTA | Leu | T | G | CGA | Arg | MIS |  |  |  |
|  | CTA | Leu | A | C | CTC | Leu | SIL |  |  |  |
|  | CTA | Leu | A | G | CTG | Leu | SIL |  |  |  |
|  | CTA | Leu | A | T | CTT | Leu | SIL |  |  |  |
|  | ***CTC*** | ***Leu*** | ***C*** | ***A*** | ***ATC*** | ***Ile*** | ***MIS*** | 0.5 | 0.5 | 0.0 |
|  | CTC | Leu | C | G | GTC | Val | MIS |  |  |  |
|  | CTC | Leu | C | T | TTC | Phe | MIS |  |  |  |
|  | CTC | Leu | T | A | CAC | His | MIS |  |  |  |
|  | CTC | Leu | T | C | CCC | Pro | MIS |  |  |  |
|  | CTC | Leu | T | G | CGC | Arg | MIS |  |  |  |
|  | ***CTC*** | ***Leu*** | ***C*** | ***A*** | ***CTA*** | ***Leu*** | ***SIL*** |  |  |  |
|  | CTC | Leu | C | G | CTG | Leu | SIL |  |  |  |
|  | CTC | Leu | C | T | CTT | Leu | SIL |  |  |  |
|  | ***CTG*** | ***Leu*** | ***C*** | ***A*** | ***ATG*** | ***Met*** | ***MIS*** | 0.5 | 0.5 | 0.0 |
|  | CTG | Leu | C | G | GTG | Val | MIS |  |  |  |
|  | CTG | Leu | C | T | TTG | Leu | SIL |  |  |  |
|  | CTG | Leu | T | A | CAG | Gln | MIS |  |  |  |
|  | CTG | Leu | T | C | CCG | Pro | MIS |  |  |  |
|  | CTG | Leu | T | G | CGG | Arg | MIS |  |  |  |
|  | CTG | Leu | G | A | CTA | Leu | SIL |  |  |  |
|  | CTG | Leu | G | C | CTC | Leu | SIL |  |  |  |
|  | ***CTG*** | ***Leu*** | ***G*** | ***T*** | ***CTT*** | ***Leu*** | ***SIL*** |  |  |  |
|  | ***CTT*** | ***Leu*** | ***C*** | ***A*** | ***ATT*** | ***Ile*** | ***MIS*** | 1.0 | 0.0 | 0.0 |
|  | CTT | Leu | C | G | GTT | Val | MIS |  |  |  |
|  | CTT | Leu | C | T | TTT | Phe | MIS |  |  |  |
|  | CTT | Leu | T | A | CAT | His | MIS |  |  |  |
|  | CTT | Leu | T | C | CCT | Pro | MIS |  |  |  |
|  | CTT | Leu | T | G | CGT | Arg | MIS |  |  |  |
|  | CTT | Leu | T | A | CTA | Leu | SIL |  |  |  |
|  | CTT | Leu | T | C | CTC | Leu | SIL |  |  |  |
|  | CTT | Leu | T | G | CTG | Leu | SIL |  |  |  |
|  | ***GAA*** | ***Glu*** | ***G*** | ***T*** | ***TAA*** | ***STOP*** | ***NON*** | 0.0 | 0.0 | 1.0 |
|  | GAA | Glu | G | A | AAA | Lys | MIS |  |  |  |
|  | GAA | Glu | G | C | CAA | Gln | MIS |  |  |  |
|  | GAA | Glu | A | C | GCA | Ala | MIS |  |  |  |
|  | GAA | Glu | A | G | GGA | Gly | MIS |  |  |  |
|  | GAA | Glu | A | T | GTA | Val | MIS |  |  |  |
|  | GAA | Glu | A | C | GAC | Asp | MIS |  |  |  |
|  | GAA | Glu | A | T | GAT | Asp | MIS |  |  |  |
|  | GAA | Glu | A | G | GAG | Glu | SIL |  |  |  |

|  | GAC | Asp | G | A | AAC | Asn | MIS | 1.0 | 0.0 | 0.0 |
| --- | --- | --- | --- | --- | --- | --- | --- | --- | --- | --- |
|  | GAC | Asp | G | C | CAC | His | MIS |  |  |  |
|  | ***GAC*** | ***Asp*** | ***G*** | ***T*** | ***TAC*** | ***Tyr*** | ***MIS*** |  |  |  |
|  | GAC | Asp | A | C | GCC | Ala | MIS |  |  |  |
|  | GAC | Asp | A | G | GGC | Gly | MIS |  |  |  |
|  | GAC | Asp | A | T | GTC | Val | MIS |  |  |  |
|  | ***GAC*** | ***Asp*** | ***C*** | ***A*** | ***GAA*** | ***Glu*** | ***MIS*** |  |  |  |
|  | GAC | Asp | C | G | GAG | Glu | MIS |  |  |  |
|  | GAC | Asp | C | T | GAT | Asp | SIL |  |  |  |
|  | ***GAG*** | ***Glu*** | ***G*** | ***T*** | ***TAG*** | ***STOP*** | ***NON*** | 0.5 | 0.0 | 0.5 |
|  | GAG | Glu | G | A | AAG | Lys | MIS |  |  |  |
|  | GAG | Glu | G | C | CAG | Gln | MIS |  |  |  |
|  | GAG | Glu | A | C | GCG | Ala | MIS |  |  |  |
|  | GAG | Glu | A | G | GGG | Gly | MIS |  |  |  |
|  | GAG | Glu | A | T | GTG | Val | MIS |  |  |  |
|  | GAG | Glu | G | C | GAC | Asp | MIS |  |  |  |
|  | ***GAG*** | ***Glu*** | ***G*** | ***T*** | ***GAT*** | ***Asp*** | ***MIS*** |  |  |  |
|  | GAG | Glu | G | A | GAA | Glu | SIL |  |  |  |
|  | GAT | Asp | G | A | AAT | Asn | MIS | 1.0 | 0.0 | 0.0 |
|  | GAT | Asp | G | C | CAT | His | MIS |  |  |  |
|  | ***GAT*** | ***Asp*** | ***G*** | ***T*** | ***TAT*** | ***Tyr*** | ***MIS*** |  |  |  |
|  | GAT | Asp | A | C | GCT | Ala | MIS |  |  |  |
|  | GAT | Asp | A | G | GGT | Gly | MIS |  |  |  |
|  | GAT | Asp | A | T | GTT | Val | MIS |  |  |  |
|  | GAT | Asp | T | A | GAA | Glu | MIS |  |  |  |
|  | GAT | Asp | T | G | GAG | Glu | MIS |  |  |  |
|  | GAT | Asp | T | C | GAC | Asp | SIL |  |  |  |
|  | GCA | Ala | G | A | ACA | Thr | MIS | 1.0 | 0.0 | 0.0 |
|  | ***GCA*** | ***Ala*** | ***G*** | ***T*** | ***TCA*** | ***Ser*** | ***MIS*** |  |  |  |
|  | GCA | Ala | G | C | CCA | Pro | MIS |  |  |  |
|  | GCA | Ala | C | G | GGA | Gly | MIS |  |  |  |
|  | ***GCA*** | ***Ala*** | ***C*** | ***A*** | ***GAA*** | ***Glu*** | ***MIS*** |  |  |  |
|  | GCA | Ala | C | T | GTA | Val | MIS |  |  |  |
|  | GCA | Ala | A | C | GCC | Ala | SIL |  |  |  |
|  | GCA | Ala | A | G | GCG | Ala | SIL |  |  |  |
|  | GCA | Ala | A | T | GCT | Ala | SIL |  |  |  |
|  | GCC | Ala | G | A | ACC | Thr | MIS | 0.66 | 0.33 | 0.0 |
|  | ***GCC*** | ***Ala*** | ***G*** | ***T*** | ***TCC*** | ***Ser*** | ***MIS*** |  |  |  |
|  | GCC | Ala | G | C | CCC | Pro | MIS |  |  |  |
|  | GCC | Ala | C | G | GGC | Gly | MIS |  |  |  |
|  | ***GCC*** | ***Ala*** | ***C*** | ***A*** | ***GAC*** | ***Asp*** | ***MIS*** |  |  |  |
|  | GCC | Ala | C | T | GTC | Val | MIS |  |  |  |
|  | ***GCC*** | ***Ala*** | ***C*** | ***A*** | ***GCA*** | ***Ala*** | ***SIL*** |  |  |  |
|  | GCC | Ala | C | G | GCG | Ala | SIL |  |  |  |
|  | GCC | Ala | C | T | GCT | Ala | SIL |  |  |  |

|  | GCG | Ala | G | A | ACG | Thr | MIS | 0.66 | 0.33 | 0.0 |
| --- | --- | --- | --- | --- | --- | --- | --- | --- | --- | --- |
|  | ***GCG*** | ***Ala*** | ***G*** | ***T*** | ***TCG*** | ***Ser*** | ***MIS*** |  |  |  |
|  | GCG | Ala | G | C | CCG | Pro | MIS |  |  |  |
|  | GCG | Ala | C | G | GGG | Gly | MIS |  |  |  |
|  | ***GCG*** | ***Ala*** | ***C*** | ***A*** | ***GAG*** | ***Glu*** | ***MIS*** |  |  |  |
|  | GCG | Ala | C | T | GTG | Val | MIS |  |  |  |
|  | GCG | Ala | G | A | GCA | Ala | SIL |  |  |  |
|  | GCG | Ala | G | C | GCC | Ala | SIL |  |  |  |
|  | ***GCG*** | ***Ala*** | ***G*** | ***T*** | ***GCT*** | ***Ala*** | ***SIL*** |  |  |  |
|  | GCT | Ala | G | A | ACT | Thr | MIS | 1.0 | 0.0 | 0.0 |
|  | ***GCT*** | ***Ala*** | ***G*** | ***T*** | ***TCT*** | ***Ser*** | ***MIS*** |  |  |  |
|  | GCT | Ala | G | C | CCT | Pro | MIS |  |  |  |
|  | GCT | Ala | C | G | GGT | Gly | MIS |  |  |  |
|  | ***GCT*** | ***Ala*** | ***C*** | ***A*** | ***GAT*** | ***Asp*** | ***MIS*** |  |  |  |
|  | GCT | Ala | C | T | GTT | Val | MIS |  |  |  |
|  | GCT | Ala | T | A | GCA | Ala | SIL |  |  |  |
|  | GCT | Ala | T | C | GCC | Ala | SIL |  |  |  |
|  | GCT | Ala | T | G | GCG | Ala | SIL |  |  |  |
|  | ***GGA*** | ***Gly*** | ***G*** | ***T*** | ***TGA*** | ***STOP*** | ***NON*** | 0.5 | 0.0 | 0.5 |
|  | GGA | Gly | G | A | AGA | Arg | MIS |  |  |  |
|  | GGA | Gly | G | C | CGA | Arg | MIS |  |  |  |
|  | GGA | Gly | G | C | GCA | Ala | MIS |  |  |  |
|  | GGA | Gly | G | A | GAA | Glu | MIS |  |  |  |
|  | ***GGA*** | ***Gly*** | ***G*** | ***T*** | ***GTA*** | ***Val*** | ***MIS*** |  |  |  |
|  | GGA | Gly | A | C | GGC | Gly | SIL |  |  |  |
|  | GGA | Gly | A | G | GGG | Gly | SIL |  |  |  |
|  | GGA | Gly | A | T | GGT | Gly | SIL |  |  |  |
|  | GGC | Gly | G | A | AGC | Ser | MIS | 0.66 | 0.33 | 0.0 |
|  | GGC | Gly | G | C | CGC | Arg | MIS |  |  |  |
|  | ***GGC*** | ***Gly*** | ***G*** | ***T*** | ***TGC*** | ***Cys*** | ***MIS*** |  |  |  |
|  | GGC | Gly | G | C | GCC | Ala | MIS |  |  |  |
|  | GGC | Gly | G | A | GAC | Asp | MIS |  |  |  |
|  | ***GGC*** | ***Gly*** | ***G*** | ***T*** | ***GTC*** | ***Val*** | ***MIS*** |  |  |  |
|  | ***GGC*** | ***Gly*** | ***C*** | ***A*** | ***GGA*** | ***Gly*** | ***SIL*** |  |  |  |
|  | GGC | Gly | C | G | GGG | Gly | SIL |  |  |  |
|  | GGC | Gly | C | T | GGT | Gly | SIL |  |  |  |
|  | GGG | Gly | G | A | AGG | Arg | MIS | 0.66 | 0.33 | 0.0 |
|  | GGG | Gly | G | C | CGG | Arg | MIS |  |  |  |
|  | ***GGG*** | ***Gly*** | ***G*** | ***T*** | ***TGG*** | ***Trp*** | ***MIS*** |  |  |  |
|  | GGG | Gly | G | C | GCG | Ala | MIS |  |  |  |
|  | GGG | Gly | G | A | GAG | Glu | MIS |  |  |  |
|  | **GGG** | **Gly** | **G** | **T** | **GTG** | **Val** | **MIS** |  |  |  |
|  | GGG | Gly | G | A | GGA | Gly | SIL |  |  |  |
|  | GGG | Gly | G | C | GGC | Gly | SIL |  |  |  |
|  | ***GGG*** | ***Gly*** | ***G*** | ***T*** | ***GGT*** | ***Gly*** | ***SIL*** |  |  |  |

|  |  |  |  |  |  |  |  |  |  |  |
| --- | --- | --- | --- | --- | --- | --- | --- | --- | --- | --- |
|  | GGT | Gly | G | A | AGT | Ser | MIS | 1.0 | 0.0 | 0.0 |
|  | GGT | Gly | G | C | CGT | Arg | MIS |  |  |  |
|  | ***GGT*** | ***Gly*** | ***G*** | ***T*** | ***TGT*** | ***Cys*** | ***MIS*** |  |  |  |
|  | GGT | Gly | G | C | GCT | Ala | MIS |  |  |  |
|  | GGT | Gly | G | A | GAT | Asp | MIS |  |  |  |
|  | ***GGT*** | ***Gly*** | ***G*** | ***T*** | ***GTT*** | ***Val*** | ***MIS*** |  |  |  |
|  | GGT | Gly | T | A | GGA | Gly | SIL |  |  |  |
|  | GGT | Gly | T | C | GGC | Gly | SIL |  |  |  |
|  | GGT | Gly | T | G | GGG | Gly | SIL |  |  |  |
|  | GTA | Val | G | A | ATA | Ile | MIS | 1.0 | 0.0 | 0.0 |
|  | GTA | Val | G | C | CTA | Leu | MIS |  |  |  |
|  | ***GTA*** | ***Val*** | ***G*** | ***T*** | ***TTA*** | ***Leu*** | ***MIS*** |  |  |  |
|  | GTA | Val | T | A | GAA | Glu | MIS |  |  |  |
|  | GTA | Val | T | C | GCA | Ala | MIS |  |  |  |
|  | GTA | Val | T | G | GGA | Gly | MIS |  |  |  |
|  | GTA | Val | A | C | GTC | Val | SIL |  |  |  |
|  | GTA | Val | A | G | GTG | Val | SIL |  |  |  |
|  | GTA | Val | A | T | GTT | Val | SIL |  |  |  |
|  | GTC | Val | G | A | ATC | Ile | MIS | 0.5 | 0.5 | 0.0 |
|  | GTC | Val | G | C | CTC | Leu | MIS |  |  |  |
|  | ***GTC*** | ***Val*** | ***G*** | ***T*** | ***TTC*** | ***Phe*** | ***MIS*** |  |  |  |
|  | GTC | Val | T | A | GAC | Asp | MIS |  |  |  |
|  | GTC | Val | T | C | GCC | Ala | MIS |  |  |  |
|  | GTC | Val | T | G | GGC | Gly | MIS |  |  |  |
|  | ***GTC*** | ***Val*** | ***C*** | ***A*** | ***GTA*** | ***Val*** | ***SIL*** |  |  |  |
|  | GTC | Val | C | G | GTG | Val | SIL |  |  |  |
|  | GTC | Val | C | T | GTT | Val | SIL |  |  |  |
|  | GTG | Val | G | A | ATG | Met | MIS | 0.5 | 0.5 | 0.0 |
|  | GTG | Val | G | C | CTG | Leu | MIS |  |  |  |
|  | ***GTG*** | ***Val*** | ***G*** | ***T*** | ***TTG*** | ***Leu*** | ***MIS*** |  |  |  |
|  | GTG | Val | T | A | GAG | Glu | MIS |  |  |  |
|  | GTG | Val | T | C | GCG | Ala | MIS |  |  |  |
|  | GTG | Val | T | G | GGG | Gly | MIS |  |  |  |
|  | GTG | Val | G | A | GTA | Val | SIL |  |  |  |
|  | GTG | Val | G | C | GTC | Val | SIL |  |  |  |
|  | ***GTG*** | ***Val*** | ***G*** | ***T*** | ***GTT*** | ***Val*** | ***SIL*** |  |  |  |
|  | GTT | Val | G | A | ATT | Ile | MIS | 1.0 | 0.0 | 0.0 |
|  | GTT | Val | G | C | CTT | Leu | MIS |  |  |  |
|  | ***GTT*** | ***Val*** | ***G*** | ***T*** | ***TTT*** | ***Phe*** | ***MIS*** |  |  |  |
|  | GTT | Val | T | A | GAT | Asp | MIS |  |  |  |
|  | GTT | Val | T | C | GCT | Ala | MIS |  |  |  |
|  | GTT | Val | T | G | GGT | Gly | MIS |  |  |  |
|  | GTT | Val | T | A | GTA | Val | SIL |  |  |  |
|  | GTT | Val | T | C | GTC | Val | SIL |  |  |  |
|  | GTT | Val | T | G | GTG | Val | SIL |  |  |  |

|  |  |  |  |  |  |  |  |  |  |  |
| --- | --- | --- | --- | --- | --- | --- | --- | --- | --- | --- |
|  | TAC | Tyr | T | A | AAC | Asn | MIS | 0.0 | 0.0 | 1.0 |
|  | TAC | Tyr | T | C | CAC | His | MIS |  |  |  |
|  | TAC | Tyr | T | G | GAC | Asp | MIS |  |  |  |
|  | TAC | Tyr | A | T | TTC | Phe | MIS |  |  |  |
|  | TAC | Tyr | A | C | TCC | Ser | MIS |  |  |  |
|  | TAC | Tyr | A | G | TGC | Cys | MIS |  |  |  |
|  | ***TAC*** | ***Tyr*** | ***C*** | ***A*** | ***TAA*** | ***STOP*** | ***NON*** |  |  |  |
|  | TAC | Tyr | C | G | TAG | STOP | NON |  |  |  |
|  | TAC | Tyr | C | T | TAT | Tyr | SIL |  |  |  |
|  | TAT | Tyr | T | A | AAT | Asn | MIS | 0.0 | 0.0 | 0.0 |
|  | TAT | Tyr | T | C | CAT | His | MIS |  |  |  |
|  | TAT | Tyr | T | G | GAT | Asp | MIS |  |  |  |
|  | TAT | Tyr | A | T | TTT | Phe | MIS |  |  |  |
|  | TAT | Tyr | A | C | TCT | Ser | MIS |  |  |  |
|  | TAT | Tyr | A | G | TGT | Cys | MIS |  |  |  |
|  | TAT | Tyr | T | A | TAA | STOP | NON |  |  |  |
|  | TAT | Tyr | T | G | TAG | STOP | NON |  |  |  |
|  | TAT | Tyr | T | C | TAC | Tyr | SIL |  |  |  |
|  | TCA | Ser | T | A | ACA | Thr | MIS | 0.0 | 0.0 | 1.0 |
|  | TCA | Ser | T | C | CCA | Pro | MIS |  |  |  |
|  | TCA | Ser | T | G | GCA | Ala | MIS |  |  |  |
|  | ***TCA*** | ***Ser*** | ***C*** | ***A*** | ***TAA*** | ***STOP*** | ***NON*** |  |  |  |
|  | TCA | Ser | C | G | TGA | STOP | NON |  |  |  |
|  | TCA | Ser | C | T | TTA | Leu | MIS |  |  |  |
|  | TCA | Ser | A | C | TCC | Ser | SIL |  |  |  |
|  | TCA | Ser | A | G | TCG | Ser | SIL |  |  |  |
|  | TCA | Ser | A | T | TCT | Ser | SIL |  |  |  |
|  | TCC | Ser | T | A | ACC | Thr | MIS | 0.5 | 0.5 | 0.0 |
|  | TCC | Ser | T | C | CCC | Pro | MIS |  |  |  |
|  | TCC | Ser | T | G | GCC | Ala | MIS |  |  |  |
|  | ***TCC*** | ***Ser*** | ***C*** | ***A*** | ***TAC*** | ***Tyr*** | ***MIS*** |  |  |  |
|  | TCC | Ser | C | G | TGC | Cys | MIS |  |  |  |
|  | TCC | Ser | C | T | TTC | Phe | MIS |  |  |  |
|  | ***TCC*** | ***Ser*** | ***C*** | ***A*** | ***TCA*** | ***Ser*** | ***SIL*** |  |  |  |
|  | TCC | Ser | C | G | TCG | Ser | SIL |  |  |  |
|  | TCC | Ser | C | T | TCT | Ser | SIL |  |  |  |
|  | TCG | Ser | T | A | ACG | Thr | MIS | 0.0 | 0.5 | 0.5 |
|  | TCG | Ser | T | C | CCG | Pro | MIS |  |  |  |
|  | TCG | Ser | T | G | GCG | Ala | MIS |  |  |  |
|  | ***TCG*** | ***Ser*** | ***C*** | ***A*** | ***TAG*** | ***STOP*** | ***NON*** |  |  |  |
|  | TCG | Ser | C | G | TGG | Trp | MIS |  |  |  |
|  | TCG | Ser | C | T | TTG | Leu | MIS |  |  |  |
|  | TCG | Ser | G | A | TCA | Ser | SIL |  |  |  |
|  | TCG | Ser | G | C | TCC | Ser | SIL |  |  |  |
|  | ***TCG*** | ***Ser*** | ***G*** | ***T*** | ***TCT*** | ***Ser*** | ***SIL*** |  |  |  |

|  | TCT | Ser | T | A | ACT | Thr | MIS | 1.0 | 0.0 | 0.0 |
| --- | --- | --- | --- | --- | --- | --- | --- | --- | --- | --- |
|  | TCT | Ser | T | C | CCT | Pro | MIS |  |  |  |
|  | TCT | Ser | T | G | GCT | Ala | MIS |  |  |  |
|  | ***TCT*** | ***Ser*** | ***C*** | ***A*** | ***TAT*** | ***Tyr*** | ***MIS*** |  |  |  |
|  | TCT | Ser | C | G | TGT | Cys | MIS |  |  |  |
|  | TCT | Ser | C | T | TTT | Phe | MIS |  |  |  |
|  | TCT | Ser | T | A | TCA | Ser | SIL |  |  |  |
|  | TCT | Ser | T | C | TCC | Ser | SIL |  |  |  |
|  | TCT | Ser | T | G | TCG | Ser | SIL |  |  |  |
|  | TGC | Cys | T | A | AGC | Ser | MIS | 0.5 | 0.0 | 0.5 |
|  | TGC | Cys | T | C | CGC | Arg | MIS |  |  |  |
|  | TGC | Cys | T | G | GGC | Gly | MIS |  |  |  |
|  | TGC | Cys | G | A | TAC | Tyr | MIS |  |  |  |
|  | TGC | Cys | G | C | TCC | Ser | MIS |  |  |  |
|  | ***TGC*** | ***Cys*** | ***G*** | ***T*** | ***TTC*** | ***Phe*** | ***MIS*** |  |  |  |
|  | ***TGC*** | ***Cys*** | ***C*** | ***A*** | ***TGA*** | ***STOP*** | ***NON*** |  |  |  |
|  | TGC | Cys | C | G | TGG | Trp | MIS |  |  |  |
|  | TGC | Cys | C | T | TGT | Cys | SIL |  |  |  |
|  | TGG | Trp | T | A | AGG | Arg | MIS | 1.0 | 0.0 | 0.0 |
|  | TGG | Trp | T | C | CGG | Arg | MIS |  |  |  |
|  | TGG | Trp | T | G | GGG | Gly | MIS |  |  |  |
|  | TGG | Trp | G | A | TAG | STOP | NON |  |  |  |
|  | TGG | Trp | G | C | TCG | Ser | MIS |  |  |  |
|  | ***TGG*** | ***Trp*** | ***G*** | ***T*** | ***TTG*** | ***Leu*** | ***MIS*** |  |  |  |
|  | TGG | Trp | G | A | TGA | STOP | NON |  |  |  |
|  | TGG | Trp | G | C | TGC | Cys | MIS |  |  |  |
|  | ***TGG*** | ***Trp*** | ***G*** | ***T*** | ***TGT*** | ***Cys*** | ***MIS*** |  |  |  |
|  | TGT | Cys | T | A | AGT | Ser | MIS | 1.0 | 0.0 | 0.0 |
|  | TGT | Cys | T | C | CGT | Arg | MIS |  |  |  |
|  | TGT | Cys | T | G | GGT | Gly | MIS |  |  |  |
|  | TGT | Cys | G | A | TAT | Tyr | MIS |  |  |  |
|  | TGT | Cys | G | C | TCT | Ser | MIS |  |  |  |
|  | ***TGT*** | ***Cys*** | ***G*** | ***T*** | ***TTT*** | ***Phe*** | ***MIS*** |  |  |  |
|  | TGT | Cys | T | A | TGA | STOP | NON |  |  |  |
|  | TGT | Cys | T | G | TGG | Trp | MIS |  |  |  |
|  | TGT | Cys | T | C | TGC | Cys | SIL |  |  |  |
|  | TTA | Leu | T | A | ATA | Ile | MIS | 0.0 | 0.0 | 0.0 |
|  | TTA | Leu | T | G | GTA | Val | MIS |  |  |  |
|  | TTA | Leu | T | C | CTA | Leu | SIL |  |  |  |
|  | TTA | Leu | T | A | TAA | STOP | NON |  |  |  |
|  | TTA | Leu | T | G | TGA | STOP | NON |  |  |  |
|  | TTA | Leu | T | C | TCA | Ser | MIS |  |  |  |
|  | TTA | Leu | A | C | TTC | Phe | MIS |  |  |  |
|  | TTA | Leu | A | T | TTT | Phe | MIS |  |  |  |
|  | TTA | Leu | A | G | TTG | Leu | SIL |  |  |  |

|  | TTC | Phe | T | A | ATC | Ile | MIS | 1.0 | 0.0 | 0.0 |
| --- | --- | --- | --- | --- | --- | --- | --- | --- | --- | --- |
|  | TTC | Phe | T | C | CTC | Leu | MIS |  |  |  |
|  | TTC | Phe | T | G | GTC | Val | MIS |  |  |  |
|  | TTC | Phe | T | A | TAC | Tyr | MIS |  |  |  |
|  | TTC | Phe | T | C | TCC | Ser | MIS |  |  |  |
|  | TTC | Phe | T | G | TGC | Cys | MIS |  |  |  |
|  | ***TTC*** | ***Phe*** | ***C*** | ***A*** | ***TTA*** | ***Leu*** | ***MIS*** |  |  |  |
|  | TTC | Phe | C | G | TTG | Leu | MIS |  |  |  |
|  | TTC | Phe | C | T | TTT | Phe | SIL |  |  |  |
|  | TTG | Leu | T | A | ATG | Met | MIS | 1.0 | 0.0 | 0.0 |
|  | TTG | Leu | T | G | GTG | Val | MIS |  |  |  |
|  | TTG | Leu | T | C | CTG | Leu | SIL |  |  |  |
|  | TTG | Leu | T | A | TAG | STOP | NON |  |  |  |
|  | TTG | Leu | T | C | TCG | Ser | MIS |  |  |  |
|  | TTG | Leu | T | G | TGG | Trp | MIS |  |  |  |
|  | TTG | Leu | G | C | TTC | Phe | MIS |  |  |  |
|  | ***TTG*** | ***Leu*** | ***G*** | ***T*** | ***TTT*** | ***Phe*** | ***MIS*** |  |  |  |
|  | TTG | Leu | G | A | TTA | Leu | SIL |  |  |  |
|  | TTT | Phe | T | A | ATT | Ile | MIS | 0.0 | 0.0 | 0.0 |
|  | TTT | Phe | T | C | CTT | Leu | MIS |  |  |  |
|  | TTT | Phe | T | G | GTT | Val | MIS |  |  |  |
|  | TTT | Phe | T | A | TAT | Tyr | MIS |  |  |  |
|  | TTT | Phe | T | C | TCT | Ser | MIS |  |  |  |
|  | TTT | Phe | T | G | TGT | Cys | MIS |  |  |  |
|  | TTT | Phe | T | A | TTA | Leu | MIS |  |  |  |
|  | TTT | Phe | T | G | TTG | Leu | MIS |  |  |  |
|  | TTT | Phe | T | C | TTC | Phe | SIL |  |  |  |
