# Supplementary material for: Use of signals of positive and negative selection to distinguish cancer genes and passenger genes
Source: eLife. 2021 Jan 11;10:e59629. doi: 10.7554/eLife.59629 (PMC7877913; doi:10.7554/eLife.59629)
Supplement: Supplementary file 25. [file elife-59629-supp25.docx]

**Supplementary file 25**

**Expected fractions of nonsense, missense and silent substitutions in the absence of selection assuming equal codon frequency and that only C>A or C>G or C>T or T>A or T>C or T>G mutations occur.**

**Amino acid mutation nucleotide substitution**

C>A C>G C>T T>A T>C T>G

**Nonsense** 0.07447 0.02128 0.05319 0.07865 0.00000 0.02247

**Missense** 0.72340 0.80851 0.61702 0.71910 0.65169 0.76404

**Silent**  0.20213 0.17021 0.32979 0.20225 0.34831 0.21348

The fractions of nonsense, missense and silent amino acid mutations of individual codons are shown in Supplementary files 18-24.
